# What counts as a voiceable concern in decisions about speaking out in hospitals: A qualitative study

**DOI:** 10.1177/13558196211043800

**Published:** 2022-01-03

**Authors:** Mary Dixon-Woods, Emma L Aveling, Anne Campbell, Akbar Ansari, Carolyn Tarrant, Janet Willars, Peter Pronovost, Imogen Mitchell, David W Bates, Christian Dankers, James McGowan, Graham Martin

**Affiliations:** 1 Health Foundation Professor of Healthcare Improvement Studies, THIS Institute, Department of Public Health and Primary Care, 12204 University of Cambridge, UK; 2 Research Scientist, Department of Health Policy and Management, 1857 Harvard TH Chan School of Public Health, Boston, MA, USA; 3 Research Associate, 572200 The NIHR Health Protection Research Unit in Healthcare Associated Infections and Antimicrobial Resistance, Imperial College London, UK; 4 Research Associate, THIS Institute, Department of Public Health and Primary Care, 12204 University of Cambridge, UK; 5 Professor of Health Services Research, Department of Health Sciences, 4488 University of Leicester, UK; 6 Honorary Visiting Fellow, Department of Health Sciences, 4488 University of Leicester, UK; 7 Chief Clinical Transformation and Chief Quality Officer, 24575 University Hospitals Cleveland, OH, USA; 8 Professor, Department of Anesthesiology and Critical Care Medicine, School of Medicine, Western Reserve University, Cleveland, OH, USA; 9 Executive Director, 104822 Research and Academic Partnerships, Canberra Health Services and Australian National University; 10 Chief, Division of General Internal Medicine, 1861 Brigham and Women’s Hospital, Boston, MA, USA; 11 Associate Chief Quality Officer, Quality and Patient Experience, 1813 Mass General Brigham, Boston, MA, USA; 12 Clinical Research Associate, THIS Institute, Department of Public Health and Primary Care, 12204 University of Cambridge, UK; 13 Director of Research, THIS Institute, Department of Public Health and Primary Care, 12204 University of Cambridge, UK

**Keywords:** Voice behaviour, qualitative research, hospitals

## Abstract

**Objectives:**

Those who work in health care organisations are a potentially valuable source of information about safety concerns, yet failures of voice are persistent. We propose the concept of ‘voiceable concern’ and offer an empirical exploration.

**Methods:**

We conducted a qualitative study involving 165 semi-structured interviews with a range of staff (clinical, non-clinical and at different hierarchical levels) in three hospitals in two countries. Analysis was based on the constant comparative method.

**Results:**

Our analysis shows that identifying what counts as a concern, and what counts as a occasion for voice by a given individual, is not a straightforward matter of applying objective criteria. It instead often involves discretionary judgement, exercised in highly specific organisational and cultural contexts. We identified four influences that shape whether incidents, events and patterns were classified as voiceable concerns: certainty that something is wrong and is an occasion for voice; system versus conduct concerns, forgivability and normalisation. Determining what counted as a voiceable concern is not a simple function of the features of the concern; also important is whether the person who noticed the concern felt it was voiceable by them.

**Conclusions:**

Understanding how those who work in health care organisations come to recognise what counts as a voiceable concern is critical to understanding decisions and actions about speaking out. The concept of a voiceable concern may help to explain aspects of voice behaviour in organisations as well as informing interventions to improve voice.

## Introduction

Those who work in health care organisations are a potentially valuable source of information about safety concerns, poor care, faulty systems or inappropriate conduct.^1,2^ However, health care workers do not always give voice to concerns.^3,4^ Their reasons for remaining silent on workplace issues include their perceptions of hierarchical unsupportive organisational climates, of the likely benefits and risks of an act of voice, including damage to relationships and being viewed negatively, and of having insufficient authority or security of employment.^5-9^ Recent work on implicit voice theories, that is, people’s socially acquired, taken-for-granted beliefs about why and when speaking up at work might be risky or inappropriate,^10^ has further deepened understanding of how people make decisions about giving voice.

It is less clear how people come to recognise concerns, given, for example, that they may be faced with multiple ambiguities about whether a problem really exists or is of a magnitude to warrant mentioning.^8,11^ Detert and Edmondson^10^ offer the concept of latent voice episodes, which refers to ‘specific instances in which a would-be speaker believes the possibility exists to speak up to someone with positional power in a face to-face context about something of importance’.^10, p. 462^ However, they do not offer an expanded account of how it is that these episodes might come to be defined as such by those in a given situation.^10^ What is understood by people as a concern to which they might give voice has remained under-explored and under-theorised, yet is likely to be important to explaining aspects of voice behaviour.

We here propose the concept of the ‘voiceable concern’ and we use data from a large number of interviews about speaking out in hospitals to identify what influences people’s identification of what counts as a voiceable concern. We propose, as a starting point, that classifying a situation, incident or pattern as a voiceable concern has two components. First, it must be recognised as a concern, and second, it must also be recognised as one that could or should be voiced. However, on their own these two simple criteria are not sufficient either for practical purposes (e.g. for those wishing to improve voice in organisations) or analytic purposes (e.g. for those seeking to theorise voice). In developing the concept further, we draw on a long-standing research tradition in social and political science that has sought to understand how particular issues come to be defined as problems, the classificatory processes involved, and the social career of problems once so defined.^12–14^ This finds that the classification of instances of even apparently straightforward constructs may turn out to be social practices as much as technical ones.^15^ When it comes to messier and more subjective situations, such as safety concerns in the health care context, what counts as an instance of something may be even more contingent. In the context of medical error*,* for example, Bosk argues that particular instances may not be ‘easily recognized and agreed upon by all concerned’.^16, p. 23^ Instead, errors are an ‘essentially contested’ concept, open to debate on each and every occasion of use.^16,17^ Bosk’s discussion of how error is understood and negotiated in practice shows how the grounds for judging an event as an error are not fixed, but rather negotiated in interaction, with meanings that are ‘fluid and flexible, highly dependent on context’.^17, p. 2^

This kind of analysis has not yet been applied to questions of voice and silence (and the spaces in between) in health care settings, nor has it been extended beyond considerations of error to the broader concept of ‘concerns’. In this study, we seek to deepen and refine the concept of a voiceable concern and to characterise the influences that may operate on recognition of such concerns both as concerns and as occasions for voice.

## Methods

We conducted a qualitative study involving semi-structured interviews in three hospitals in two countries (sites 1–3) selected as case studies;^18,19^ see Table 1 for summary characteristics of the participating sites, reproduced from another publication elsewhere.^20^ The selection of sites was initially pragmatic: one organisation, having experienced a serious problem involving patient harm, commissioned a study to understand how to improve voice by examining practices of speaking and listening within its hospitals. Following initial data collection and analysis, two other sites were chosen purposefully to extend the analysis and to test the transferability of constructs to other contexts: one organisation, in the same country as the first and with some similar characteristics (a prestigious teaching hospital), had undertaken a programme of cultural enhancement that included a focus on practices of voice; the other was in a different high-income country.

**Table 1.** Summary characteristics of the three participating sites.

	Site 1	Site 2	Site 3
National context			
Country classification	High-income country	High-income country	High-income country
Health care system categorisation	Private health system (privately funded, market-regulated, privately provided)	National health insurance (taxation-funded, government-regulated, privately provided)	Private health system (privately funded, market-regulated, privately provided)
Organisational attributes			
Organisation characteristics	Integrated academic health system including medical school, acute and community hospitals, and primary care	Academic hospital including medical school, acute hospital and community services	Academic medical centre covering a wide range of acute specialties, part of a wider integrated health system
Annual admissions (to nearest 10k)	100,000	70,000	50,000
Inpatient beds (to nearest 500)	2500	500	1000
Employment model	Non-medical staff directly employed; physicians predominantly independent or university-employed	Most staff directly employed	Non-medical staff directly employed; physicians predominantly independent contractor
Organisational context			
Prevailing infrastructure for voicing concerns	Range of formal mechanisms including incident-reporting system for behavioural as well as clinical matters. Confidential hotline. Policies codifying behavioural expectations and responsibilities. Guidance on how to escalate concerns	Range of formal mechanisms including incident-reporting system. Central group responsible for responding to cross-departmental concerns identified. Drop-in sessions for staff with concerns. Policy for appropriate escalation of concerns raised	Range of formal mechanisms including incident-reporting system. Formally specified remedial interventions to clinicians where behaviour has caused problems. Training in human-systems interaction
Additional relevant background	Widely publicised, recent case of long-running abuse by a prominent physician	Widely publicised case of poor quality care and mistreatment of whistleblowers	Long-standing emphasis on just culture approach to incidents and concerns

Table also appears in Wu et al.^20^

An email including an information sheet was sent by heads of department to invite senior leaders (e.g. departmental chairs, executives, board members) and those at point-of-care or ‘front line’ (e.g. physicians, nurses, technical and administrative staff, and environmental and housekeeping staff) to take part in a confidential interview. Interested individuals could respond directly to the research team through a confidential website. We interviewed everyone who responded to the invitation for whom we were able to arrange an interview; no further sampling within the responders was undertaken. We were unable to calculate the number of people who received initial invitations owing to the way emailing was handled by participating departments.

Semi-structured interviews were conducted using a topic guide that included questions about how participants would raise concerns if they became aware of situations or practices that they felt might not support patient safety. The guides were modified slightly between sites to take account of the specifics of each site. All interviews were digitally recorded. At site 1, interviews were conducted by GM and JW; all interviews at sites 2 and 3 were conducted by ELA. All interviewers had a background in psychology or social science, and were experienced qualitative researchers with no relationship to the study sites. The data for the study were collected between 2014 and 2016.

Interviews were recorded upon consent. Recordings were transcribed verbatim, and transcripts were anonymised and identifying details removed. Digital recordings were deleted after transcription and no link retained between transcripts and participants. Full transcripts were not shared with the participating hospitals.

Data analysis was undertaken by AC, ELA, JM and MDW based on the constant comparative method.^21^ We used sensitising constructs from the relevant social science literature. A selection of interviews at each site was open-coded to develop an initial coding frame, which was applied to subsequent interview transcripts, and iteratively developed as new codes were identified. This process continued until all transcripts were analysed and no further new codes added to the coding frame. NVivo 10 software was used to manage the coding and analysis process.

### Ethics approval

This study was submitted to ethics review system at each of the participating hospitals. At two sites, it received approval (approval number not disclosed for reasons of confidentiality). At the other site, it was deemed quality improvement and was exempted from Institutional Review Board approval; the study team nonetheless used a consent procedure at all sites.

## Results

We conducted 165 interviews across the three sites (Table 2), from an initial 329 individuals who expressed interest in taking part. In the presentation of our findings, we attribute quotations to either frontline staff (FL), who were mostly clinicians, or senior leader (SL) participants, who included some clinicians with dual responsibilities. Details of some quotations have been disguised to ensure confidentiality, for example, by changing minor details of the scenarios or individuals described.

**Table 2.** Responses to invitation and interviews conducted across the three sites.

	Responses to invitation	Interviews conducted	Interviews with mid/senior leaders	Interviews with frontline personnel
Site 1	118	67	20	47
Site 2	78	47	16	31
Site 3	133	51	21	30
Total	329	165	57	108

We were able to characterise four influences on whether people classified something as a voiceable concern: their certainty both that something is wrong and that it is an occasion for them to give voice; whether the concern centres on a system issue or a conduct issue; how far the concern is seen as being forgivable; and the extent of local normalisation of the issues relating to the concern. Critically, we found that what counted as a voiceable concern was not simply a function of the concern itself, but was also powerfully determined by whether the person who noticed the concern felt it could and should be voiced by them, in the given situation.

### Certainty that something is wrong and an occasion for voice by the person who noticed it

We found that some situations were easily and straightforwardly recognised by participants as sources of concern. Examples included where unequivocal risk of immediate harm to the patient was present, or when an egregious injury or violation had already taken place. Participants reported little hesitation about these events and their own responsibility to give voice to them. But many moments or patterns lacked this straightforward character and were fraught with ambiguity.

The big ones are ones that you really kind of pick up right away, are usually the ones that are quite black and white, and there’s some greyer ones, and you’re like maybe I should have said something or maybe I should have done something. *(Site 3, FL)*

Possible opportunities to speak were more complicated when they related to an emerging or established pattern rather than to a specific, easily defined incident. Some participants described a generalised sense that things were ‘not right’ that did not resolve into a specific instance. They gave accounts of patterns of mishaps, behaviours or signals that, while individually minor, created a gestalt of something badly wrong. Also challenging were perceived or anticipated hazards: something had yet to go wrong, but it appeared that it might.

It is not that anything specific has happened yet but you can foresee that something could happen […] we had a lab report that was delayed. We didn’t get the results for two days and although it was within normal limits, it was a test that we kind of monitor […] had it been too high or too low and we did not get the results for two days, it could have had a negative impact so that I reported. It could have been a problem everywhere. *(Site 1, FL)*

Identifying something as a voiceable concern was, for participants, intimately linked to the quality of the evidence underlying their concern, whether they felt qualified to make a well-informed judgement, and the extent to which they felt they could justify the reasons for their concern. Participants described multiple situations where they felt discomfort, but insufficient certainty to determine whether the concern was legitimately a matter of concern requiring voice. In such instances, people might feel they lacked the clinical, technical or procedural knowledge to make a call.

That [clinical situation] is not an area that I feel completely comfortable in, so I was a little bit uncertain about my ground clinically [...] I haven’t raised it directly with that person, and I haven’t raised it with their supervisor either. *(Site 2, FL)*

Judgements about what counted as a concern were particularly difficult where the possible concern related to the potential violation of an objective and observable standard (‘procedural neglect’), as opposed to situations which were related to the amount of pain or distress (physical or emotional) a patient was suffering (‘caring neglect’).^22^ The latter were seen as more difficult to judge and voice.

I didn’t speak out because I wasn’t sure. But [the doctor] was putting in a chest drain on a patient, and I had seen chest drains put in before, and generally, you know, they do prove to be a bit discomforting to the patient [...] but this particular patient seemed to be in excruciating pain as he was putting it in. And I kind of thought ‘oh, maybe he’s not doing it right’, being that I’ve never seen a patient in so much pain, and then he sort of wouldn’t stop [...] At the time I kind of thought ‘ah do I say something?’ But I didn’t, because I really, I’ve never put one in in my life, I wouldn’t know, it’s really not my expertise. *(Site 2, FL)*

When describing such situations, participants noted that there might be uncertainty over who could claim jurisdiction over the definition of what should be an occasion for concern, reflecting Hughes’s recognition of how a particular group may come to believe that it alone ‘fully understands the technical contingencies’ and therefore jealously guards its right to make the judgement about mistakes.^23, p. 323^

[A doctor] was actually syringing the ears out of this patient, and I [nurse] was doing a few other things in the room. And I was just watching him, because I was quite concerned at how he was doing it, and his patient was actually expressing on a number of occasions quite serious pain, and he kept going, and he just kept dismissing it, saying ‘oh, it’s just the wax that’s just causing the pain, or it’s just that’. And she was actually like ‘no no no, you need to stop, that actually really really hurts’ [...] I found it a really difficult position to be in, because sometimes you just, I guess in some ways there’s still that kind of, ‘well I’m the doctor, I know better’. *(Site 2, FL)*

### The concern centres on a system issue or a conduct issue

Another key influence affecting the recognition of a voiceable concern was the extent to which the focus of a concern related to an identifiable individual or team issue, a system issue or a hybrid of both. Participants reported that they generally (although not universally) found it easier to identify system issues as voiceable concerns compared to situations where a specific individual appeared to be at fault. System issues, such as IT problems, test ordering or medication processes, were seen to be factual in character and tractable to improvement. Most of all, these issues were not seen as being blameworthy*.*

Bringing up system issues, people are more comfortable with that. There’s not as much value judgment laden in that. *(Site 1, FL)*

But concerns related to behaviour or conduct were seen as trickier, potentially harder to judge and more discomfiting to evidence and to articulate. Behaviours that caused potential concern included responding to disagreements with obstruction or anger, rudeness or using inappropriate language, bullying, harassment or discrimination, abuse of power, and refusal to listen to the wishes of patients or carers. Particularly difficult were subtle matters of tone, language, and respect.

Most people kind of know that you can’t be yelling, throwing things, the really egregious stuff. But you know, is there a curtness in tone, you know, or is there a little sarcasm, that you bringing up that concern reflects your lack of clinical understanding, or you know, you’re not as smart as me or you’re not as expert as I am. *(Site 3, FL)*

### Extent to which the concern is seen as being forgivable

Both Freidson and Bosk distinguished between errors or mistakes that might be seen as ‘normal’^17,24, p. 131^ and which may be deemed forgivable, and ‘deviant’ errors or mistakes, which may be seen as arising from ‘negligence, ignorance, or ineptitude’.^24, p. 131^ Participants reported a similar distinction in relation to concerns. Identifying what counted as a voiceable concern was especially fraught when it required a determination of whether behaviour was unacceptable or unprofessional as opposed to ‘just’ unpleasant, or excusable given the circumstances.

In the first instance I would expect the executive to establish the facts, have a direct conversation with the clinician involved, and then it very very much depends on, on whether or not this is a repeat offender, or whether it was just somebody who was having a bad day and there was a good reason why, or it was acceptable. *(Site 2, SL)*

Theoretically, if someone does a procedure and they don’t do it correctly, I mean that’s reportable, right? But I know for a fact that that [procedure that wasn’t done correctly] wasn’t reported, because you know, he is learning, and it’s a training programme, you know, next time it’ll be better, sort of, you know, it didn’t harm the patient, but it may not help the patient. So I don’t know. If you really wanted to follow the letter of the law, you know, you probably should have done a safety report on that. *(Site 3, FL)*

We found that judgements about giving voice were also moderated by consideration of whether a concern appeared to be part of a pattern of behaviour: concerns thought to be one-off lapses uncharacteristic of the individual involved could be to be deemed more forgivable.

It’s so hard, sometimes, in these situations to think—to not rationalise it away. ‘Oh, they work a hundred hours. They’re just tired. I should accept it.’ You know what I mean? As opposed to saying, ‘You know, that’s just wrong.’ *(Site 1, SL)*

### Extent of local normalisation of the issues relating to the concern

Participants reported widespread uncertainty about voicing concerns where the issue giving rise to concern appeared highly normalised in the environment in which it was occurring.

I was talking to some of the clinicians about [a safety incident]. One in particular was telling me that when he first came here […] he sort of found it odd that our process was looser here in terms of being able to request and obtain blood for transfusion than it was in other places where he’d worked […] His comment was that once you’re sort of engrained here for a little while you just accept ‘OK, this is the way that the policy works here, this is the way the process goes’ and you sort of just start to live with it and accept it and that’s the way that it is. *(Site 1, SL)*

The acceptance of poor standards and the inability to recognise problems happened not just in relation to systems and processes, but also in relation to poor conduct, such as disrespectful, aggressive behaviour towards colleagues, in a process consistent with what Vaughan describes as the ‘normalisation of deviance’.^25^ It was enabled not only by their becoming ‘the way things are done around here’ but also by cultural reluctance to tackle difficult problems head-on, especially if such problems were already entrenched. In some cases, the situations were so discomfiting, and the consequences of raising them so fearful, that people simply chose not to confront them at all. Grants of forgiveness (sometimes self-serving, in the sense that they absolved candidate speakers of responsibility for action) were sometimes made to those engaging in poor behaviours. This sometimes allowed poor conduct to persist unchecked, and therefore become increasingly normalised.

I spoke to the nursing team leader and I said ‘something’s wrong here [...] and she said ‘ah yes, that’s just Dr Blah Blah, he does that’. And I said ‘can you tell him that he can’t be doing that, because I see no justification for doing that’. But that’s just tolerated because that’s how he is, I think that’s unacceptable [...] and when I asked that she speak to him she sort of indicated that ‘no, well that’s just how he is’, she wouldn’t approach him. *(Site 2, FL)*

Normalisation also contributed to misplaced trust, by reducing scepticism and alertness to the possibility that poor conduct or practice might be occurring.^26^

So clinicians have a tendency to trust each other. ‘Everybody’s like me. They’re honest, they’re hard-working and they’re not using drugs.’ Which was involved in this case. So it was a culture of trust. There wasn’t a lot of scepticism […] we have a culture of trust so we don’t often see things […] behaviour sometimes has to get extreme before someone reports*. (Site 1, SL)*

## Discussion

Failures of voice in health care settings are highly consequential, not least in their implications for patient safety and quality of care. This study, involving a large number of interviews across three hospitals in two countries, suggests the value and utility of the concept of a ‘voiceable concern’ and indicates that if people are to speak out about issues of quality and safety in health care organisations, concerns must be recognised both as concerns and as occasions for voice by those who have noticed them. Our analysis shows that how people come to recognise concerns as concerns, and their judgements about whether those concerns should give rise to voice, are not straightforward matters involving objective criteria. Decisions about what counts as a concern that should and/or could be voiced are contextually embedded, and distinctions between the nature of a concern and the entitlement or opportunity to speak are not always easily made. We identified four influences on people’s judgements about what counted as a voiceable concern: certainty about whether something is wrong and is an occasion for voice by a candidate speaker, system versus conduct issues, forgivability and normalisation.

The concept of voiceable concern can inform fresh understanding of the barriers to voice in organisations. Although known inhibitors of voice, such as fear of risks and consequences, reluctance to engage in demanding and irksome processes, and potential for disrupted relationships remain crucially important,^5–9^ we propose that renewed emphasis is needed on the prior challenge of recognising an occasion for voice: that is, identifying a concern, and classifying it as one that should give rise to voice by a candidate speaker. Our findings to some extent build on the concept of latent voice episodes, defined as occasions when a ‘would-be speaker believes the possibility exists to speak up […] about something of importance’.^10, p. 462^ However, we found that what counted as a ‘possibility’ and as ‘something of importance’ were highly contingent and dynamic, and that such episodes were not necessarily confined to specific instances.

We propose that what counts as a voiceable concern requires scrutiny in its own right. We suggest that voiceable concerns are best understood as a form of social practice: they are not naturally occurring, consensually understood events. We further propose that voiceable concerns are, to use Bosk’s term, ‘essentially contested’.^16,17^ While there is general agreement on the phenomenon, what counts as a particular instance, the issue of possible concern and its interpretation, as well as the responsibility, entitlement and opportunity to speak, is often clouded with uncertainty. This does not mean that intervention to improve behaviours in relation to voiceable concerns is futile. One way of approaching these challenges is to recognise the voiceable concern as a type of problem definition process^27^ that requires shared understanding of the features of such concerns and a shared vocabulary that allows them to be expressed and building confidence in individuals about their judgements and their entitlements to speak on the basis of those judgements.

Acting on these insights requires more than just a codified set of criteria by which the eligibility of concerns should be judged. Rather, it requires a collectively agreed understanding of what constitutes a voiceable concern, together with wider organisational reflection on the range of contextual influences that can affect both their identification and their being voiced. As our study shows, what comes to be classified as an occasion for voice is powerfully affected by wider organisational and cultural influences, including expectations, standards and norms^28,29^ and, more broadly, the fit of an episode into wider patterns of organisational behaviour. All of these are tractable to change, for example, through leadership providing clear direction on acceptable standards of conduct and practice, by minimising tolerance of breaches of community standards, and by creating forums where people can share their experiences and reflections to reach shared understanding.^19,30^

### Strengths and limitations

This study has a number of strengths in addressing the need for greater understanding of the particular messages that can potentially be voiced by health care workers,^8^ including its relatively large sample size across three different sites and its inclusion of clinicians, non-clinicians, and individuals in different positions in the hospital leadership hierarchy. It also has a number of limitations. We were unable to test for theoretical saturation (because further theoretical sampling was not possible), and thus cannot be certain whether we were able to access the full range of views in the participating organisations. We also, for reasons of confidentiality, collected only very limited information on the characteristics (including job title) of participants, and thus could not conduct analysis of whether views varied by role. Future work could usefully evaluate the degree to which our conceptual model of a voiceable concern is robust to these features. Reliance solely on interviews rather than observations or other supporting data may bring the possible risk of over-emphasis on conscious decisions, over less obvious or even unknowable antecedents of voice. Further, we could not formally assess participants’ openness and honesty in interviews. The generalisability and transferability of the findings will need to be tested in further settings, especially as the three hospitals were in high-income countries.

## Conclusions

Understanding what counts as a voiceable concern is essential to supporting voice in health care organisations. Our findings highlight the extent to which the recognition of a concern that should give rise to voice goes beyond the immediate facts of the matter and takes place in the context of both longer term patterns and socially informed judgements of culpability and forgivability, shaped and guided by multiple organisational and cultural influences as well as individual judgements. These findings have important theoretical and practical implications.
